# Non-faradaic electrochemical impedimetric profiling of procalcitonin and C-reactive protein as a dual marker biosensor for early sepsis detection

**DOI:** 10.1016/j.acax.2019.100029

**Published:** 2019-10-03

**Authors:** Ambalika Sanjeev Tanak, Badrinath Jagannath, Yashaswee Tamrakar, Sriram Muthukumar, Shalini Prasad

**Affiliations:** a Department of Bioengineering, The University of Texas at Dallas, Richardson, TX, 75080, USA; b EnLiSense LLC, 1813 Audubon Pondway, Allen, TX, 75013, USA

**Keywords:** Electrochemical impedance spectroscopy, Procalcitonin, C-reactive protein, Non-faradaic, Dual marker biosensor, Sepsis

## Abstract

In this work, we demonstrate a robust, dual marker, biosensing strategy for specific and sensitive electrochemical response of Procalcitonin and C-reactive protein in complex body fluids such as human serum and whole blood for the detection of sepsis. Enhanced sensitivity is achieved by leveraging the physicochemical properties of zinc oxide at the electrode-solution interface. Characterization techniques such as SEM, EDAX, AFM, FTIR and fluorescence microscopy were performed to ensure a suitable biosensing surface. The characteristic biomolecular interactions between the target analyte and specific capture probe is quantified through unique frequency signatures using non-faradaic electrochemical impedance spectroscopy (EIS). The developed biosensor demonstrated a detection limit of 0.10 ng mL^−1^ for PCT in human serum and whole blood with an R^2^ of 0.99 and 0.98 respectively. CRP demonstrated a detection limit of 0.10 μg mL^−1^ in human serum and whole blood with an R^2^ of 0.90 and 0.98 respectively. Cross-reactivity analysis demonstrated robust selectivity to PCT and CRP with negligible interaction to non-specific biomolecules. The novel aspect of this technology is the ability to fine-tune individual biomarkers response owing to the optimal frequency tuning capability. The developed biosensor requires an ultra-low sample volume of 10 μL without the need for sample dilution for rapid analysis. We envision the developed dual marker biosensor to be useful as a sepsis-screening device for prognostic monitoring.

## Highlights

• Ultra-sensitive, specific, low volume biosensor developed for rapid screening of host response biomarkers to detect sepsis.
• EIS captures the interfacial changes within the electrical double layer due to the binding interaction of PCT & CRP.
• Enhanced sensor response with a LOD of 0.10 ng mL^−1^ for PCT and 0.10 μg mL^−1^ for CRP and an R^2^ of 0.99 in human serum & blood.
• Biomolecular frequency tuning using electrochemical impedance spectroscopy enables multi-marker detection.
• Developed dual marker biosensor demonstrates specific response towards target biomolecules.

## Graphical abstract

**Figure:**
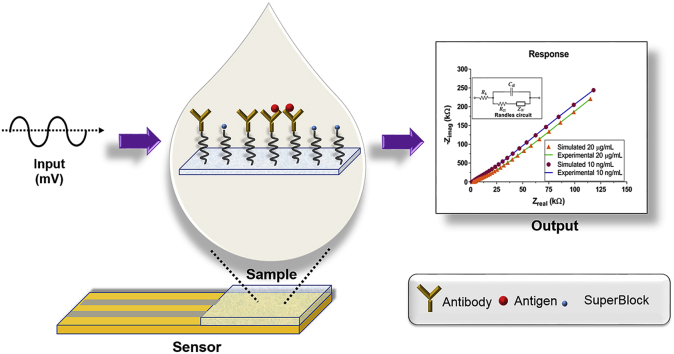

## 1 Introduction

Over the last decade, despite significant medical advancements in prevention, diagnosis, and treatment, infectious diseases remain amongst the top three causes of death worldwide according to the World Health Organization (WHO) [1]. The body’s inordinate response to an infection leads to a life-threatening medical emergency condition known as sepsis, which affects more than 30 million people globally [2]. For sepsis, every hour of delayed treatment increases the rate of mortality by 8% [3]. Timely diagnosis with rapid treatment methodology has been reported to improve the chances of preventing adverse complications, and thus, reduce mortality rate [4,5]. The clinical appearance of an infectious disease mirrors the interaction between the host and the microorganism [6]. An effective method to scan for infection is by monitoring biomarkers responsible for host immune response, which unveils the severity of sepsis. However, relying on a single biomarker to determine sepsis can lead to misdiagnosis, as sepsis is a result of multiple complication of an infection [7]. In response to an infection, the body releases multiple triggers, which impairs regular blood flow and leads to blood clots and leaky blood vessels. This deprives the organ from necessary nutrients and results in organ damage. In severe cases, blood pressure drops drastically, heart weakens and patient spirals into septic shock. The patient requires immediate and accurate diagnosis at this crucial stage. An approach for successful prognosis includes integrating a combination of an early and a late onset biomarker for achieving a patient’s comprehensive sepsis profile. The Food and Drug Administration (FDA) has recently approved Procalcitonin (PCT) and C-reactive protein (CRP) for diagnosing and monitoring sepsis in the clinical domain [8]. Given the significance of multi-marker detection, we have devised a unique strategy to encompass the diagnostic value of PCT and CRP to identify sepsis at an early stage. This strategy would encourage clinicians to design effective patient treatment plans based on the clinical profile built with disease prognosis.

PCT is 116 amino acids long protein with a molecular weight of 14.5 kD. The presence of PCT in healthy individuals is negligible (<0.05 ng mL^−1^) and escalates within 2–4 h during the onset of an infection thus, acts as an effective indicator [9]. The levels peak at 6–8 h and can surge up to 5000-fold in patients with severe sepsis [[10], [11], [12]]. PCT has a half-life of 24 h, thus, concentrations normalize quickly with recovery, which adds further clinical value in disease prognosis. Furthermore, lower PCT values can assist clinicians to discontinue empiric antibiotics in patients under treatment [13]. The second effective biomarker, CRP, is a 205 amino acid long protein with a molecular weight of approximately 24 kD [14]. Due to the excessive rise in CRP levels during an acute inflammation, this biomarker is used to indicate the presence of significant inflammatory or infectious disease, notably for sepsis [15]. Typically, the presence of CRP is detected 12–24 h after the onset of an infection and peak concentration rises 1000-fold and remains elevated up to 3–7 days [16,17]. Thus, CRP being a late onset biomarker while, PCT being an early onset biomarker serve as substantial biomarkers for sepsis detection. However, the current clinical methods rely on blood cultures as the gold standard. Routine blood cultures can take up to 5 days to identify disease pathogens [18]. These laboratory methods are cumbersome, provide delayed results and may require multiple analyses. Moreover, these tests require large patient sample volumes (1–5 mL) which further carry the risk of contamination. Despite the clinical value of laboratory methods, lack of sensitivity and specificity remain a major shortcoming [19,20]. Studies have shown that only 73% of the 629 episodes of bacteremia could be identified with a single blood culture and concluded that up to 4 sets of cultures may be required for accurate identification, excluding contaminants [21]. Furthermore, blood cultures are not rapid enough to aid the decision of therapeutic interventions at the onset of sepsis. Traditional blood cultures take 1–3 days to increase bacterial concentration to the detectable limit for molecular diagnostic testing. As a result, broad-spectrum treatment approach is adopted that may fail to target the correct microbe effectively. This could harm the patient via antimicrobial toxicity, and may contribute to evolution of drug-resistant microbes [22,23].

Point-of-care (POC) biosensors offer a fast and simple approach to overcome the existing shortcomings of laboratory-based tests. POCs allow physicians to take quick decisions to determine the course of treatment. In recent years, research groups have demonstrated POC sensors for the detection of sepsis by monitoring PCT and CRP levels. Most of these use optical surface plasmon resonance (SPR) or faradaic-based method, which require additional sample preparation that may not be feasible for providing rapid response. For instance, Park’s research group demonstrated detection of PCT by functionalizing synthetic peptides on gold electrodes and measured a detection limit of 12.5 ng mL^−1^ using faradaic approach of cyclic voltammetry and EIS [24]. Sener et al. achieved a limit of detection of 9.9 ng mL^−1^ using SPR with a response time of about 1 h for analyte in phosphate buffered saline (PBS) and simulated blood plasma [25]. Our research group has also previously shown the detection of PCT with other infectious markers with a detection limit of 0.10 ng mL^−1^ [26]. However, the sensor requires high sample volume and analyzes signal response using a single frequency for all the analytes, which could lead to cross talk during signal transduction. Similarly, Meyer MH et al. [27] used SPR for the detection of CRP and achieved a limit of detection of 2 μg mL^−1^ with a response time of approximately 1 h. Although, the reported response time using SPR is an improvement from traditional methods, it is still insufficient for rapid diagnosis. Furthermore, Songjaroen [28] and Gupta et al. [29], employed redox-based faradaic EIS to detect CRP in serum and PBS samples. However, redox dependent biosensors rely on the oxidation-reduction reaction to generate a measurable signal. This also requires additional sample preparation. Additionally, most of these reported biosensors focus on single biomarker detection, which may be insufficient for accurate diagnosis of sepsis as described previously. To overcome these shortcomings, we have developed a rapid, label-free, point-of-care biosensor with a dual marker approach to detect sepsis. The developed sensing platform comprises of two sensors on a single polyimide substrate. Each sensor is individually functionalized with a capture probe specific to PCT or CRP allowing simultaneous detection. The key features of the developed biosensor include shorter response time (<15 min), lower sample volumes (∼10 μL), sensitive and specific response with multiplexing capabilities. This was achieved using a novel sensing strategy by leveraging the advantages of a unique semiconducting material in conjunction with optimal frequency modulation using non-faradaic electrochemical impedance spectroscopy. The characteristic of this biosensor to detect low concentrations with improved sensitivity can aid in providing more information on the recovery post-treatment. In comparison with the previously described work, here in, we demonstrate a frequency tuning principle for simultaneous dual marker detection by using semiconductor active sensing electrode, thus preventing crosstalk during signal transduction. Furthermore, this sensor requires ultra-low sample volume of 10 μL for detection of each analyte.

Semiconducting materials like zinc oxide (ZnO) offer remarkable functional and morphological properties that enhances sensitivity for transducing physicochemical changes with biomolecular binding in electrochemical biosensors [30]. ZnO’s high isoelectric point (IEP∼9.5) enables stable immobilization of biomolecules with lower IEP through electrostatic interaction. This property of ZnO allows the biosensor to retain biological activity on its surface and facilitates bio functionality. Typically, biomolecules have reduced IEP as compared to ZnO at physiological pH which makes them negatively charged. Therefore, biomolecules can be readily immobilized on a positively charged ZnO through a strong electrostatic interaction. Furthermore, the chemical stability of ZnO plays a vital role in maintaining conducive environment for biomolecules in complex body fluids [31,32]. Additionally, ZnO’s crystal structure and surface polarities can be fine-tuned to enhance the electrical transfer properties that makes it suitable for electrochemical biosensing.

Typically, most of the electrochemical biosensors are DC based techniques such as chronoamperometry, cyclic voltammetry and square wave voltammetry. These methods require high concentration of redox labels to measure the signal response with additional sample preparation. Moreover, the signal response is achieved through oxidation and reduction of the redox molecule that often requires high input voltage bias. On the contrary, non-faradaic electrochemical impedance spectroscopy (EIS) is a powerful technique to capture subtle changes of the binding interaction at the electrode-solution interface without the need of a redox molecule [33,34]. Thus, EIS facilitates a non-destructive approach with a small-applied AC voltage over a frequency sweep allowing assay conjugations at the surface to remain unaffected. EIS is an excellent technique to understand charge modulation at the electrode-electrolyte interface [35].

The uniqueness of this work lies in the holistic approach of developing a unique frequency signature based multi-marker screening platform for early detection of sepsis using non-faradaic EIS.

## 2 Materials and methods

### 2.1 Reagents & materials

Thiol based cross-linker molecule dithiobis (succinimidyl propionate) (DSP) along with its solvent dimethyl sulfoxide (DMSO) were purchased from Thermo Fisher Scientific Inc (MA, USA). Stock concentration of the PCT monoclonal antibody and antigen were procured from Sigma Aldrich (MO, USA) where aliquots of required concentrations were prepared and stored at −20 °C until further use. Stock concentration of CRP monoclonal antibody along with antigen were ordered from Abcam (MA, USA). Pooled human serum, Phosphate buffered saline (PBS) and Superblock were purchased from Thermo Fisher scientific (MA, USA). Whole blood in EDTA additive tubes were procured from Carter BloodCare (TX, USA). All reagents purchased were of analytical grade and were used without any further purification.

### 2.2 ATR-IR spectroscopy

The infrared spectra collected demonstrating the functionalization of the target antibody onto the thiolated ZnO surface was captured using Thermo Scientific Nicolete iS-150 FTIR in Attenuated Total Reflectance (ATR) mode. The tool was equipped with a KBr window and deuterated triglycine sulphate (DTGS) detector. For this study, a Harrick VariGATR sampling stage with a 65° germanium crystal was used. The ATR-IR samples were prepared on a glass substrate with ZnO identical to the deposition parameters of the sensor substrate. Mimicking the sensor immunoassay (described in section 2.4), the surface was immobilized with DSP. This was followed by incubating PCT and CRP antibodies in PBS separately on the DSP functionalized ZnO surface. Prior to any functionalization, the glass substrate was cleaned thoroughly with DI water. The spectra were collected with a resolution of 4 cm^−1^ for 256 scans in a wavelength range of 4000 cm^−1^ to 600 cm^−1.^

### 2.3 Fluorescence imaging

The qualitative analysis of the binding of DSP to the ZnO surface was done by attaching a fluorophore molecule, Rhodamine 123 (Sigma Aldrich,MO), to the DSP cross-linker. 100 μL of 10 mM DSP was incubated on a ZnO coated glass slide for 90 min. After the DSP was incubated, a PBS wash was performed to remove any unbound DSP. 200 μL of 1 mM Rhodamine 123 solution was drop casted onto the substrate and incubated for 2 h. Images were captured by using a Zeiss confocal microscope with an excitation at 509 nm specific to Rhodamine 123. The first image was captured with the glass slide completely covered with Rhodamine 123 and the second image was captured after a PBS wash was performed to remove excess Rhodamine 123 molecules that had not bound to the DSP linker molecule. A control sample (without DSP) and DSP functionalized fluorophore was used to characterize the functionalization of the cross-linker through fluorescence imaging. All the images were analyzed using ImageJ to determine the intensity of fluorescence per area of the ZnO surface which corresponds to the binding of Rhodamine 123 to the DSP linker. By quantifying the fluorescence of Rhodamine 123, we were able to determine how well DSP is bound to the ZnO surface. To demonstrate the specificity of Rhodamine 123 to DSP, a control image was taken. A ZnO deposited glass slide was incubated with Rhodamine 123 for 30 min and an image was taken. PBS wash was performed to remove any excess Rhodamine 123 and follow up image was taken.

### 2.4 Immunoassay functionalization

A standard thin film fabrication technique was used to fabricate gold interdigitated electrodes on a flexible polyimide substrate purchased from Dow corning (MI, USA). A 100 nm thin film of ZnO was deposited on the gold interdigitated electrodes using RF-magnetron sputter tool. Prior to the deposition, the substrate was thoroughly cleaned with isopropyl alcohol (IPA), acetone and DI water to remove any impurities. The sensor platform comprises of two sensors on a single polyimide substrate spaced sufficiently apart. The sensors were separated using a biocompatible encapsulant made from polydimethylsiloxane (PDMS) for fluid confinement and to prevent cross-contamination over the electrode region. DSP cross-linker of 10 μL at 10 mM concentration dissolved in DMSO was incubated on each sensor for 90 min in dark at room temperature to activate effective thiol functionalization. DSP is an amine-reactive cross-linker with NHS-ester reactive ends on either arm held by a disulfide bond. NHS esters react with primary amines of antibodies to form stable amide bonds. After DSP incubation, PBS wash was performed to remove any unbound DSP. Each sensor was individually functionalized with capture probe specific to either PCT or CRP. 10 μL of 10 μg mL^−1^ PCT antibody and 20 μg mL^−1^ CRP antibody were incubated for 90 min. After antibody immobilization, the sensor surface was washed thrice with PBS to remove any unbound antibody and impedance response was measured. 10 μL of Superblock, a PBS blocking buffer, was added on the sensor and incubated for 15 min to hydrolyze any unbound linker sites. Blank buffer (serum/whole blood) was then added and measured to account for the inherent response of the buffer matrix as a control. PCT concentrations spiked in human serum were added sequentially from lower concentrations to higher concentrations in the range of 0.01 ng mL^−1^ to 10 ng mL^−1^. Similar protocol was repeated for PCT spiked in whole blood. CRP concentrations were measured from 0.01 μg mL^−1^ to 20 μg mL^−1^ in human serum and 0.01 μg mL^−1^ to 10 μg mL^−1^ in whole blood. Each spiked sample was incubated for 15 min prior to taking a measurement. Specific signal threshold (SST) line for PCT and CRP in the calibrated dose response study was defined by calculating: 3*S.D_blank_ + Mean_blank_. Limit of detection was determined as the first measureable concentration measured beyond the SST.

### 2.5 Electrochemical impedance spectroscopy (EIS)

In this work, we have used non-faradaic EIS for developing a label-free dual marker biosensor for the detection of PCT and CRP in serum and whole blood. All impedance measurements were performed using a Gamry Reference potentiostat from Gamry Instruments (PA, USA) with a 10 mV AC input over a frequency range of 1 Hz to 1 MHz. The impedance data represented is collected for n = 3 measurements unless specified.

## 3 Results and discussion

**Fig. 1:**
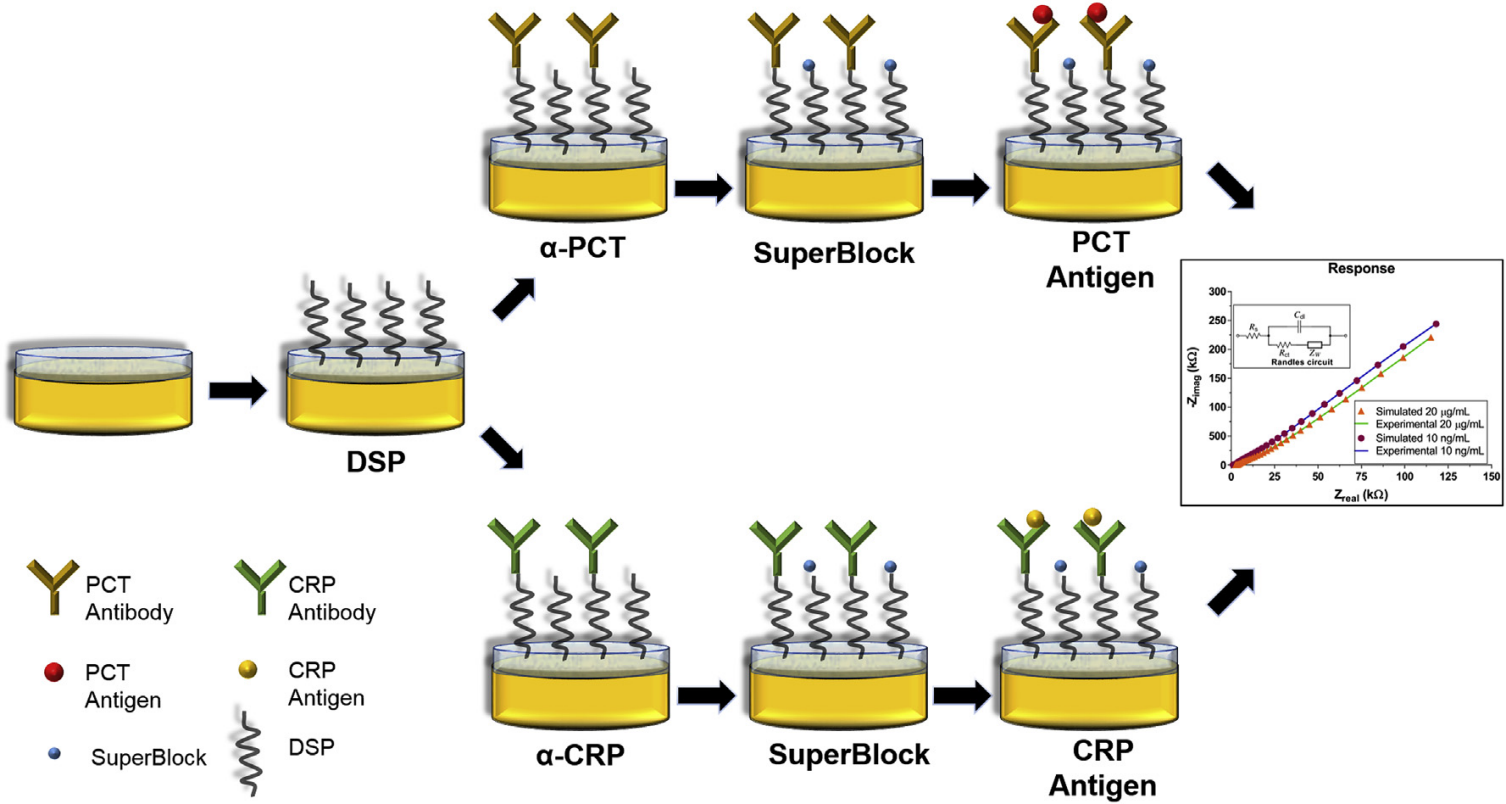
Schematic representation of the immunoassay building protocol for dual marker biosensing platform.

This work highlights the key aspects of identifying unique biomolecular signature as a combination of an early and late onset biomarker for sepsis detection. Fig. 1 schematic illustrates the immunoassay building protocol for the dual marker biosensing platform to detect sepsis. The electrode surface of the biosensor was characterized to establish uniform and stable response suitable for biosensing. Post evaluation, the surface binding chemistry was established to capture target biomolecules of PCT & CRP through affinity binding mechanism. The optimized immunoassay was further characterized to validate for selective and sensitive electrochemical response for the dual marker biosensing platform. The robust electrochemical response of PCT and CRP in complex biofluids such as human serum and whole blood were tested using EIS based on the affinity binding mechanism.

### 3.1 Characterization of semiconducting thin film electrode surface

**Fig. 2:**
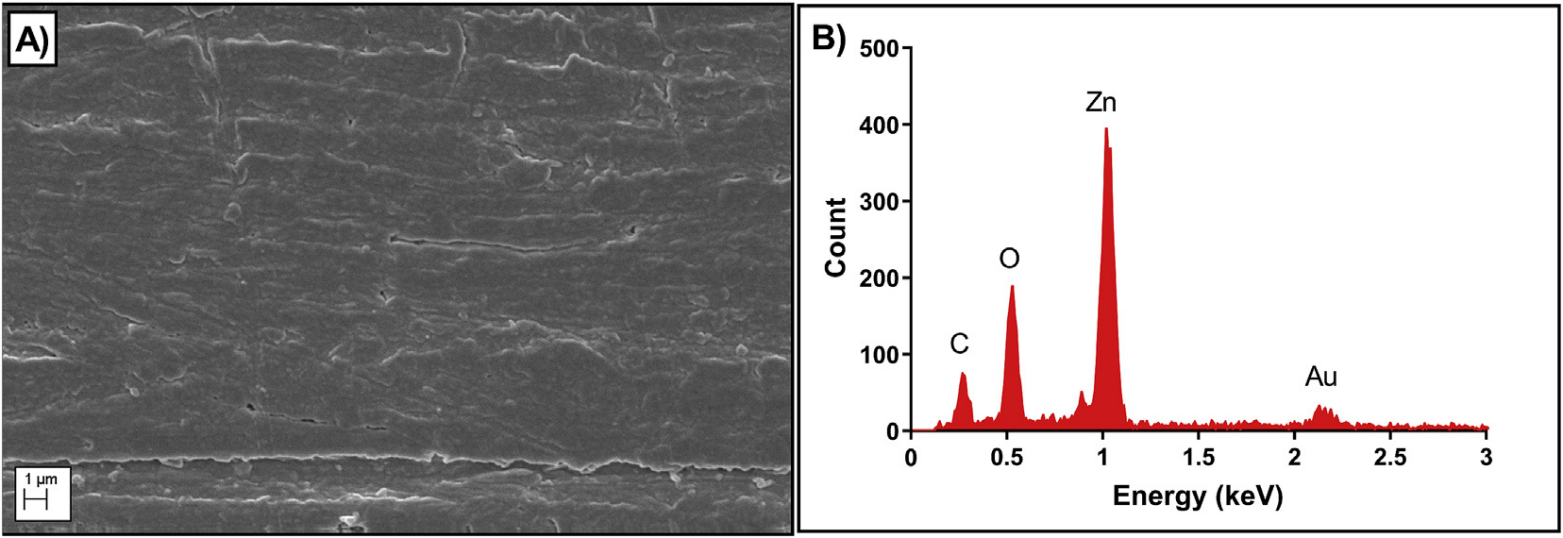
A) SEM image of the uniform deposition of ZnO thin film on the sensor platform. (B) EDAX spectra of the sensing platform representing chemical composition for the region represented in the SEM image.

The ZnO deposited thin film on the electrode surface was characterized using SEM and EDAX. Fig. 2A represents SEM image of the uniform deposition of ZnO thin film on the sensor platform with a thickness of 120 ± 20 nm. Fig. 2B represents the EDAX spectra of the sensing platform for the region represented in the SEM image. The carbon peak observed in Fig. 2B signifies the elemental composition of the polyimide substrate representing the hydrocarbon side chains. The presence of gold electrodes is confirmed by the M shell of Au peak observed at 2.125 keV. The peak observed at energy level of 1 keV corresponds to the L shell of Zinc. The oxygen peak observed at energy level 0.525 keV represents the k shell of the oxide layer of ZnO. The observed EDAX results confirms the presence of gold sensing electrode with a uniform ZnO deposition on the polyimide substrate. Furthermore, AFM was used to measure the surface roughness of the semiconducting ZnO thin film. The AFM image of the ZnO deposited film represents root mean square roughness value (Raq) of 4.57 nm (Supplementary Fig. 1). The low surface roughness value further confirms uniform deposition of ZnO thin film on the sensing electrode suitable for biosensing.

### 3.2 Evaluating surface binding chemistry using fluorescence imaging and ATR-IR analysis

**Fig. 3:**
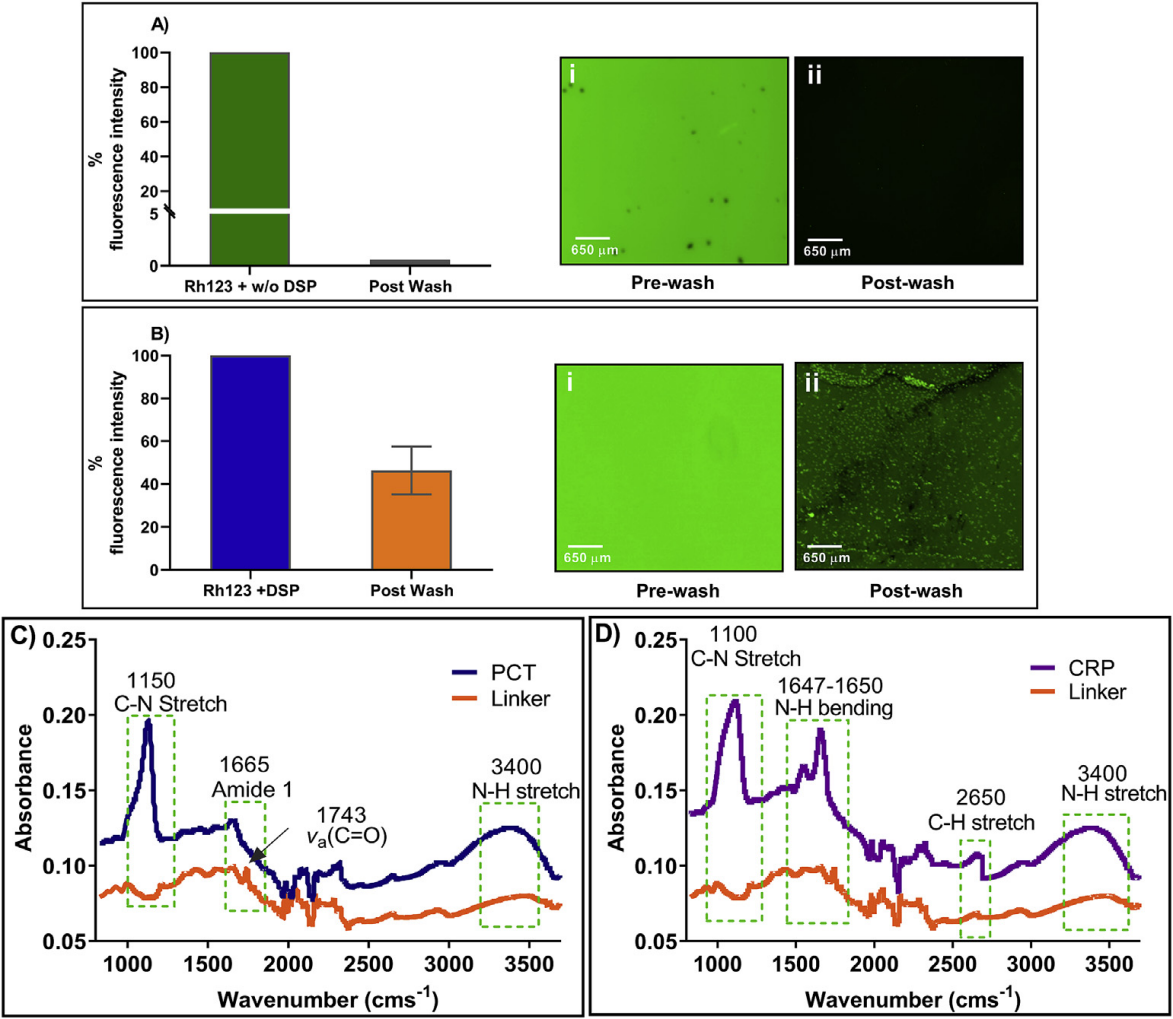
A) Fluorescence microscopy image of the sensor surface (i) pre-wash and (ii) post-wash without DSP cross-linker. (B) Fluorescence microscopy image of the modified sensor surface (i) pre-wash and (ii) post-wash with DSP cross-linker. (C) FTIR spectra of PCT and linker immobilized on ZnO modified surface. (D) FTIR spectra of CRP and linker immobilized on ZnO modified surface.

Fluorescence imaging was performed to validate binding efficiency of DSP on ZnO modified thin film surface using Rhodamine 123 as a fluorescent molecule. The functional group of Rhodamine 123 binds to the NHS ester termination of DSP linker, emitting fluorescence when excited at 509 nm. Fig. 3A and B demonstrate fluorescence microscopy analysis of the modified sensor surface pre and post wash. Surface coverage was evaluated by calculating fluorophore intensity post wash that accounts for binding of DSP. As seen in Fig. 3A, high background signal is seen due to the presence of high fluorophore concentration prior to the wash step. DI water was used to perform 3x wash to remove any residual unbound Rhodamine 123. As demonstrated in Fig. 3A (ii), the control sample did not emit fluorescence. This signifies that fluorophore binding did not occur on the ZnO thin film surface post wash step due to the absence of DSP linker molecule, thus, validating the control sample. On the contrary, fluorescence observed post wash step in Fig. 3B (ii) indicates binding of Rhodamine 123 to DSP functionalized ZnO surface. The fluorescence intensity was calculated by measuring the density of fluorescent pixels for each captured image using ImageJ. Total fluorescence was calculated by subtracting background fluorescence of the image to obtain the corrected level of fluorescence. The percent intensity was calculated by comparing the intensity post wash to the pre-wash slide intensity by using the equation (i) and (ii).

Total fluorescence = Integrated Density- (Area x Background Mean) equation (i)

Percent fluorescence= ((Total fluorescence of pre-wash slide-Total fluorescence of post wash slide)/ total fluorescence of pre-wash slide) x 100 equation (ii)

Surface coverage of DSP linker on ZnO layer is confirmed as a result of high fluorescence pixel intensity attributed to improved binding of Rhodamine 123 in the presence of DSP linker. This further validates the ZnO-thiol binding chemistry with better surface coverage of DSP binding to ZnO surface. Post visual confirmation using fluorescence imaging of the stable linker binding mechanism, the sensor surface was evaluated for its electrochemical stability.

ATR-IR analysis was performed to characterize the binding interaction of the capture probe antibody with thiol bound DSP linker between 1000 and 3500 cm^−1^ as represented in Fig. 3C and D. In Fig. 3C, the peak observed at 1316 cm^−1^ indicates symmetric C–N–C stretch of DSP [36]. The peak observed at 1743 cm^−1^ indicates the presence of free carboxylic acid in DSP. The peak observed at 1780 cm^−1^ demonstrates the presence of symmetric carbonyl stretch of NHS ester in DSP. These results confirm DSP conjugation on the ZnO modified surface. Antibody conjugation to DSP occurs by breaking of C–O bond within the NHS ester of DSP, called as aminolysis. Amine-reactive NHS ester reacts with primary amine of the antibody to form a stable amide bond. This phenomenon is seen by the disappearance of peak at 1743 cm^−1^ in the linker spectra and appearance of amide 1 peak at 1665 cm^−1^ in the PCT spectra. Additionally, broad N–H stretch at 3400 cm^−1^ and C–N stretch of amine at 1150 cm^−1^ confirms that PCT antibody was successfully bound to DSP immobilized linker substrate. Similarly, in Fig. 3D, C–N stretch observed at peak 1100 cm^−1^ along with N–H stretch observed at 3400 cm^−1^ confirms the presence of CRP antibody. Furthermore, disappearance of peak at 1743 cm^−1^ due to cleaving of C–O bond of NHS ester in DSP linker spectra and appearance of N–H bending of primary amide at 1650 cm^−1^ along with secondary amide peak at 1550 cm^−1^ of the CRP spectra indicates the conjugation of CRP antibody to DSP functionalized ZnO surface [37].

### 3.3 Electrochemical characterization of the immunoassay using electrochemical impedance spectroscopy

**Fig. 4:**
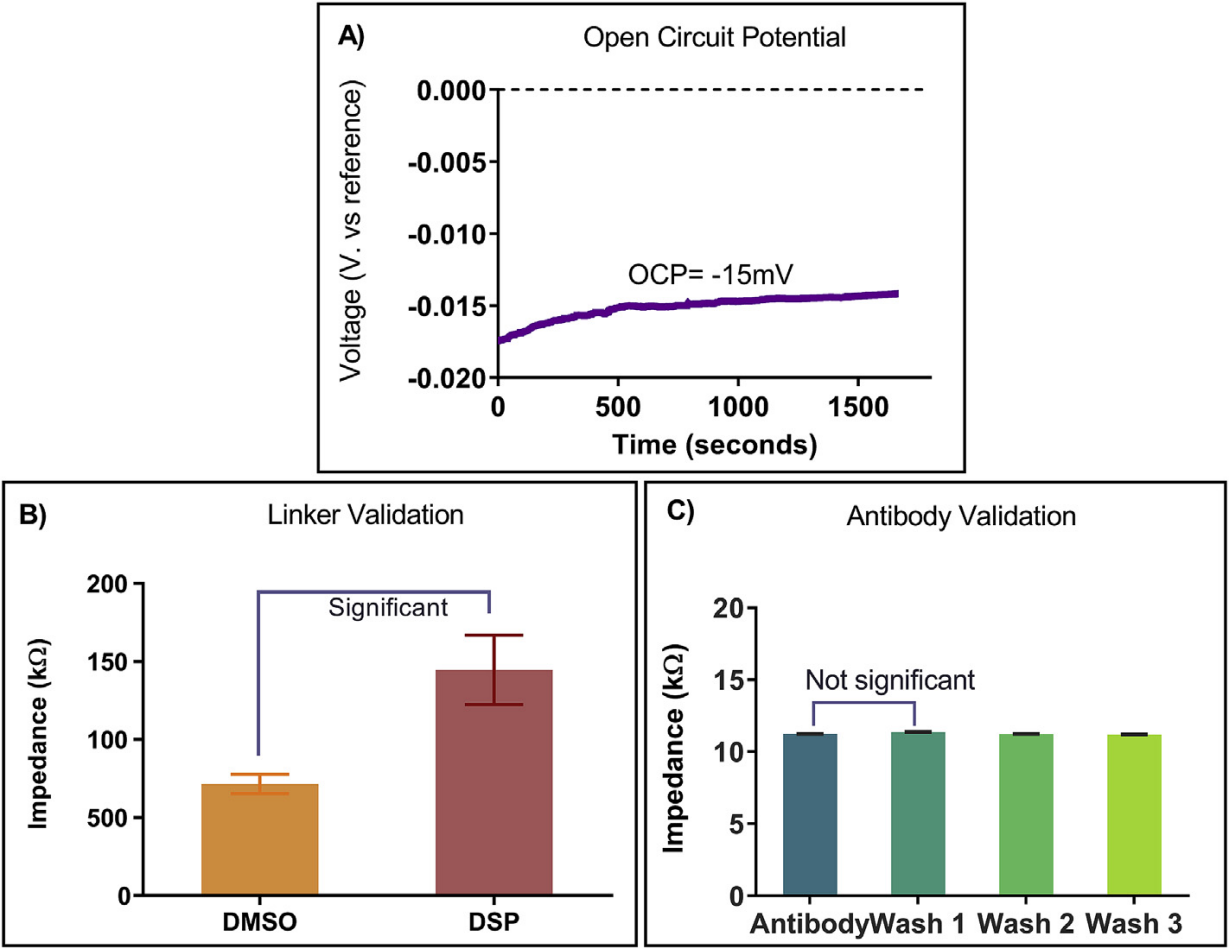
A) Electrochemical characterization of the biosensing platform with the measurement of Open circuit potential. (B) DSP linker validation to assess binding conjugation. (C) Control study to evaluate antibody binding stability.

Electrochemical characterization of the sensing electrode was evaluated by performing open circuit potential (OCP) to assess electrode stability. Open circuit potential measures the voltage difference between the reference and working electrode in the absence of applied current. OCP provides a valuable insight of the thermodynamic stability of the electrode material participating in the electrochemical response. Potential drift during open circuit potential measurement is attributed to an unstable electrode [38]. Typically, open circuit measurements are in millivolts range and stabilize within 30 min [38]. As seen in Fig. 4A, the OCP of the developed sensing electrode stabilizes at −15mV within 8 min. This signifies that the developed electrode is stable for providing electrochemical response.

The developed sensor was characterized for the baseline impedance response corresponding to each of the assay functionalization steps. Fig. 4B shows the impedance response of DMSO (∼71 kΩ) as a control followed by a significant increase in impedance post DSP conjugation (∼144 kΩ). The increase in impedance observed for DSP cross-linker is due to the known characteristic resistive behavior of DSP in DMSO [39]. To further confirm successful DSP conjugation onto the electrode surface, multiple wash steps with DMSO were performed. No significant change between the wash steps were observed (Data not included) thus, validating successful cross-linking of DSP on the electrode surface.

**Fig. 5:**
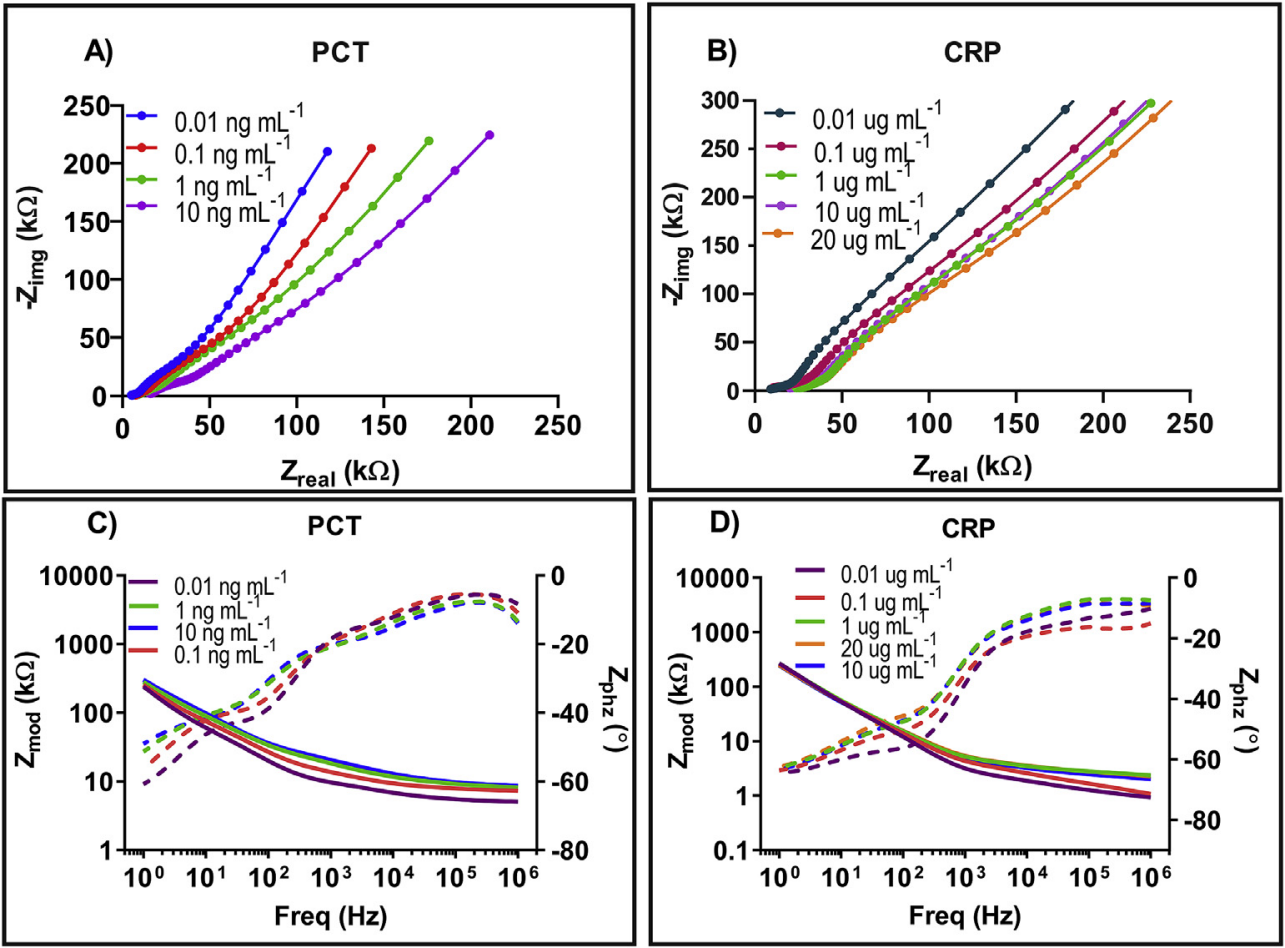
A) Nyquist plot representing impedance measurement at each concentration of PCT in human serum and (C) Bode magnitude and phase plot for impedance measured at every PCT concentration in human serum. (B) Nyquist plot representing change in impedance of CRP in human serum. (D) Bode magnitude and phase plot for impedance measured at every CRP concentration in human serum.

After successful conjugation of DSP linker on the sensing electrode, the sensor platform was evaluated for stable antibody functionalization. The impedance response was measured to be ∼11 kΩ as seen in Fig. 5C. The insignificant (p > 0.05) impedance response after several PBS wash steps confirms stable antibody functionalization to the DSP immobilized electrode surface. Furthermore, this is an electrochemical confirmation to the previously tested antibody validation seen in ATR-IR results in Fig. 3.

### 3.4 Analytical performance of the developed dual marker biosensor

The electrochemical performance of the developed biosensor was captured using non-faradaic EIS technique for the detection of PCT and CRP in human serum and whole blood. EIS is a powerful and sensitive technique which captures the electrochemical interactions at the functionalized electrode surface [40]. The stable covalent linker binding interaction helps to firmly secure the capture antibody to provide accurate biosensing response. After successfully validating the conjugation of antibody to the sensor surface through optical fluorescence microscopy, ATR-IR spectroscopy and electrochemical analysis, the specific binding interaction of the dual biomarker panel were tested in complex biofluids such as human serum and whole blood.

The electrochemical performance of the functionalized biosensor was characterized by measuring the impedance response starting from the lowest concentration to the highest concentration for the specific biomarker. The impedance results can be plotted as a Nyquist and Bode plot for PCT spiked in human serum in the range from 0.01 ng mL^−1^ to 10 ng mL^−1^ as shown in Fig. 5A and C respectively. Impedance was measured over a frequency spectrum of 1 Hz to 1 MHz for n = 3 replicates.

In a typical non-faradaic Nyquist plot, the absence of a redox label eliminates the parameters associated with electron transfer such as charge transfer resistance (Rct) and Warburg impedance by becoming infinite. This is represented by the large incomplete semicircle contributed due to the extremely slow electron transfer step which is not followed by the typical diffusion tail [41,42]. Therefore, in a non-faradaic electrochemical system, the imaginary part of impedance is inversely proportional to the electrical double layer capacitance. Furthermore, the binding interaction between the capture antibody and the target biomolecule at the electrode surface creates a charge perturbation. This phenomenon can be observed in Fig. 5A where decrease in imaginary impedance is observed with increase in the concentration of PCT. This can be leveraged to the capacitive change caused as a result of antibody-antigen binding complex within the EDL. The change in the dielectric permittivity causes a change in the double layer capacitance [43,44]. Therefore, the decrease in impedance can be interpreted as a result of increase in PCT protein concentration. The dominant capacitive behavior can further be observed in the Bode magnitude and phase plots in the region from −40° to −65° in Fig. 5C. Additionally, higher surface concentration of PCT resulted in decrease in the real impedance value. This can be attributed to the accumulation of biomolecules within the EDL resulting in the changes in conductivity of solution. Furthermore, maximum signal to noise ratio was observed at 10 Hz which was selected to analyze calibrated dose response of PCT in human serum and whole blood. Fig. 5D demonstrates a similar response for CRP spiked in human serum from 0.01 μg mL^−1^ to 20 μg mL^−1^. A dose dependent decrease in imaginary impedance indicates change in capacitance due to binding of CRP antibody antigen complex. Maximum signal to noise ratio was observed at 100 Hz which was used to compute calibrated dose response curve for CRP in human serum and whole blood. Figs. S2 and S3 in supplementary represents the Nyquist and Bode plots for PCT in whole blood. Similarly, Figs. S4 and S5 in supplementary represents the Nyquist and Bode plots for CRP in whole blood.

**Fig. 6:**
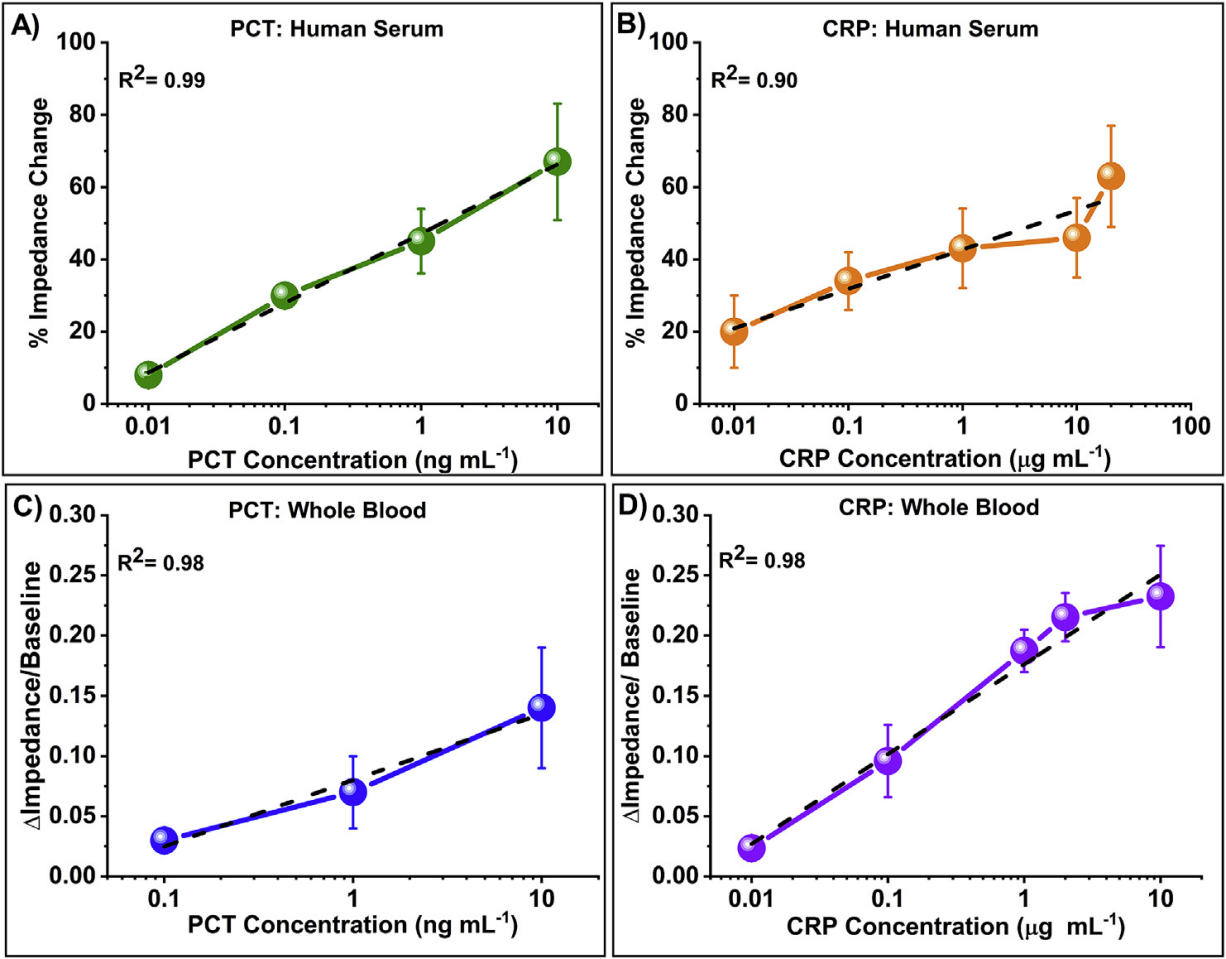
A) Calibration dose response of PCT in human serum represented as percentage change in impedance. (B) Calibration dose response of CRP in human serum represented as percentage change in impedance. (C) Calibration dose response of PCT in whole blood represented as ratio change in impedance with respect to baseline. (D) Calibration dose response of CRP in whole blood represented as ratio change in impedance with respect to baseline.

Fig. 6 represents specific calibrated dose response for the dual marker biosensor platform for PCT and CRP in human serum and whole blood. Fig. 6A represents the sensors response to varying concentrations of PCT as percentage change in impedance from the baseline (PBS measurement after blocking step) in human serum. Maximum signal response was observed at 10 Hz and was used to calculate change in impedance for PCT. The percentage change in impedance for PCT spiked in human serum between 0.01 ng mL^−1^ to 10.00 ng mL^−1^ varied from 8% to 67%. Limit of detection (LOD) for PCT in human serum was found to be 0.10 ng mL^−1^ with the coefficient of determination R^2^ of 0.99. Fig. 6C illustrates similar dose dependent response for PCT spiked in whole blood. The ratio change in impedance varies from 0.03 to 0.14 with an R^2^ of 0.98. Detection limit for PCT in whole blood was 0.10 ng mL^−1^, which lies within the physiologically relevant range.

The calibration response of the dual marker biosensor to CRP in human serum is represented in Fig. 6B over a concentration range of 0.01 μg mL^−1^ to 20 μg mL^−1^. EIS allows measuring across a spectrum of frequencies allowing to choose the optimal operating frequency with the maximum signal response. For CRP in human serum, 100 Hz was selected as the optimal operating frequency, which demonstrated enhanced signal response. Percentage change in impedance at 100 Hz varied from 20% to 63% with an R^2^ of 0.90 and detection limit of 0.10 μg mL^−1^. Extremely small AC input potential (10 mV) polarizes the electrode-electrolyte interface, causing a change in impedance due to charge perturbation. Impedance change provides information about the physio-chemical interactions between the analyte and receptor that is immobilized on the surface [45]. The dose dependent change in impedance is a contribution owing to the capacitive binding interaction of antibody-antigen interactions [46]. Furthermore, the biosensors efficacy was evaluated in whole blood as shown in Fig. 6D. Maximum ratio change in impedance was calculated to be 0.23 with a detection limit of 0.10 μg mL^−1^ and an R^2^ value of 0.98. The dynamic range of CRP in whole blood lies within the physiologically relevant range of 0.01 μg mL^−1^ to 10 μg mL^−1^.

**Fig. 7:**
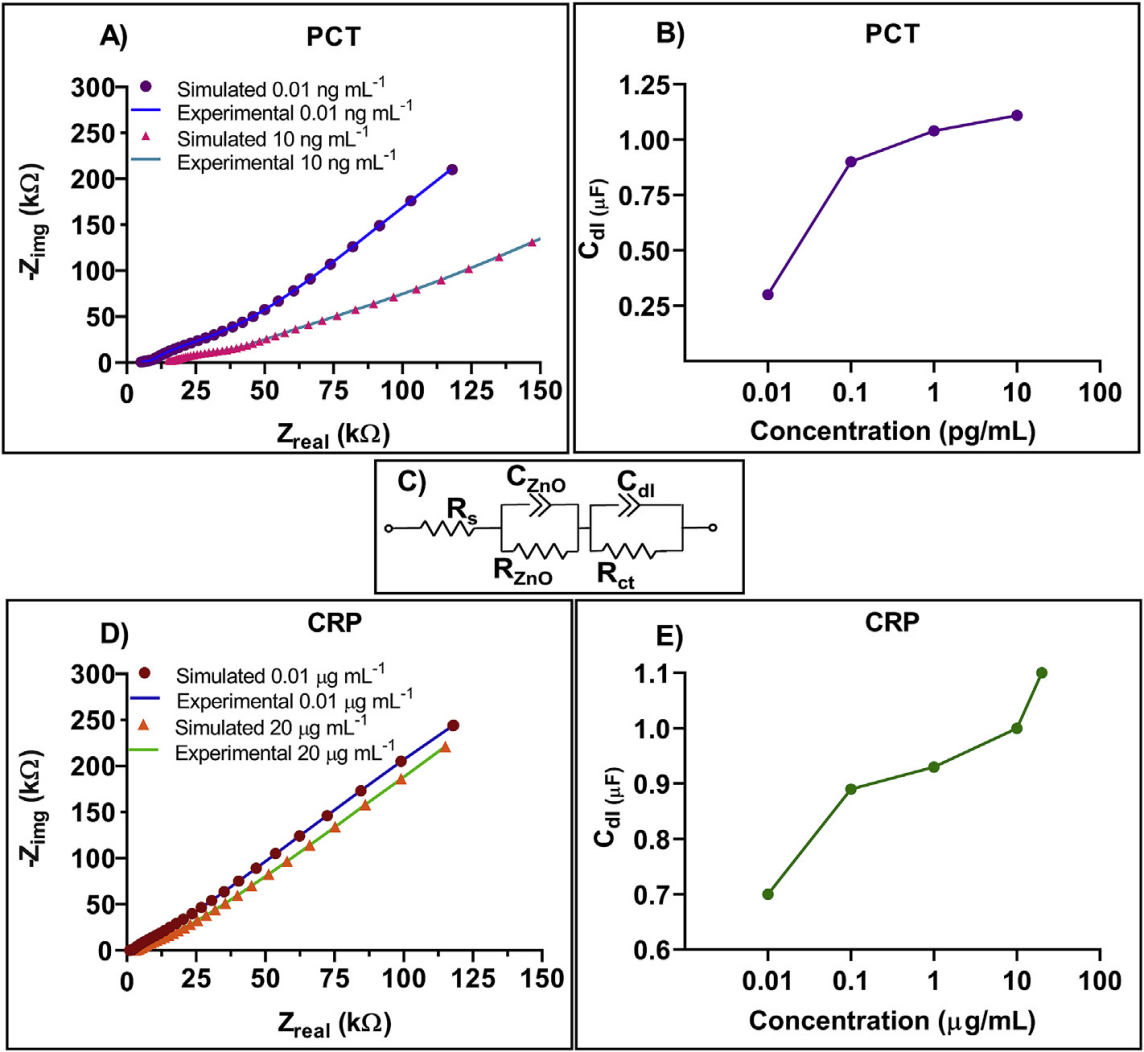
A) Experimental and circuit fit Nyquist plot of PCT at 0.01 ng mL^−1^ and 10 ng mL^−1^ concentration in human serum. (B) Capacitance values extracted for every concentration of PCT in human serum. (C) Randles equivalent circuit (D) Experimental and circuit fit Nyquist plot of CRP at 0.01 μg mL^−1^ and 20 μg mL^−1^concentration in human serum. (E) Capacitance values extracted for every concentration of CRP in human serum.

In EIS, apart from studying the individual impedance response, it allows extraction of valuable information to characterize the electrode-electrolyte system interface further in terms of conductivity, dielectric constants and capacitance. This is achieved by modelling a Randles equivalent circuit that is a combination of resistors and capacitors that mimics the electrochemical behavior electrode-solution interface. The binding interactions that occur at the electrode-electrolyte interface can further be attributed to the specific element. Typically, when a capture probe functionalized electrode encounters a target biomolecule present in an ionic buffer, the ions are adsorbed at the interface forming an electrical double layer (EDL). The bulk portion of the electrolyte lying outside the EDL contributes to the solution resistance (R_s_) in series with the parallel combination of R_ZnO_ and C_ZnO_. At lower frequency, this combination along with another RC circuit represents the charge-transfer resistance (R_ct_) and double layer capacitance (C_dl_) dominated by the interfacial binding interactions. Fig. 7 represents the circuit fit parameters extracted from the biosensing response. Fig. 7A demonstrates the experimentally obtained impedance spectra for PCT at 0.01 ng mL^−1^ and 10 ng mL^−1^ and the corresponding circuit-fit graph extracted using the equivalent circuit model shown in Fig. 7C. The changes within EDL is indicated by the semicircle represented in the low frequency region as a parallel combination of R_ct_ with C_dl_. R_s_ is represented at very high frequency which contributes to the solution resistance. The overlap observed for the experimental data with circuit fit data signifies a good least squares fit with a very Chi-Squared coefficient of 0.0005 for PCT. Fig. 7B represents the double layer capacitance values extracted for varying dose concentrations. The increase in C_dl_ with increasing dose concentrations validates the capacitive binding effect at the electrode interface occurring due to PCT antibody and protein interaction of the biosensing platform. It is observed that the capacitance increases with increase in concentration and thus, validating the decrease in impedance due to binding as described earlier. Similarly, Fig. 7D represents the fit results for experimental impedance spectra of CRP in human serum for 0.01 μg mL^−1^ and 20 μg mL^−1^ over the complete frequency spectrum. C_dl_ values extracted from the circuit fit parameters demonstrate a dose dependent increase with increase in CRP concentration in human serum. This change in capacitance at the biosensing interface is proportional to the change in CRP concentration, thus, indicating antibody-antigen binding interaction occurring at the EDL.

### 3.5 Selectivity and specificity response of the electrochemical dual marker biosensor for detection of PCT and CRP in human serum

**Fig. 8:**
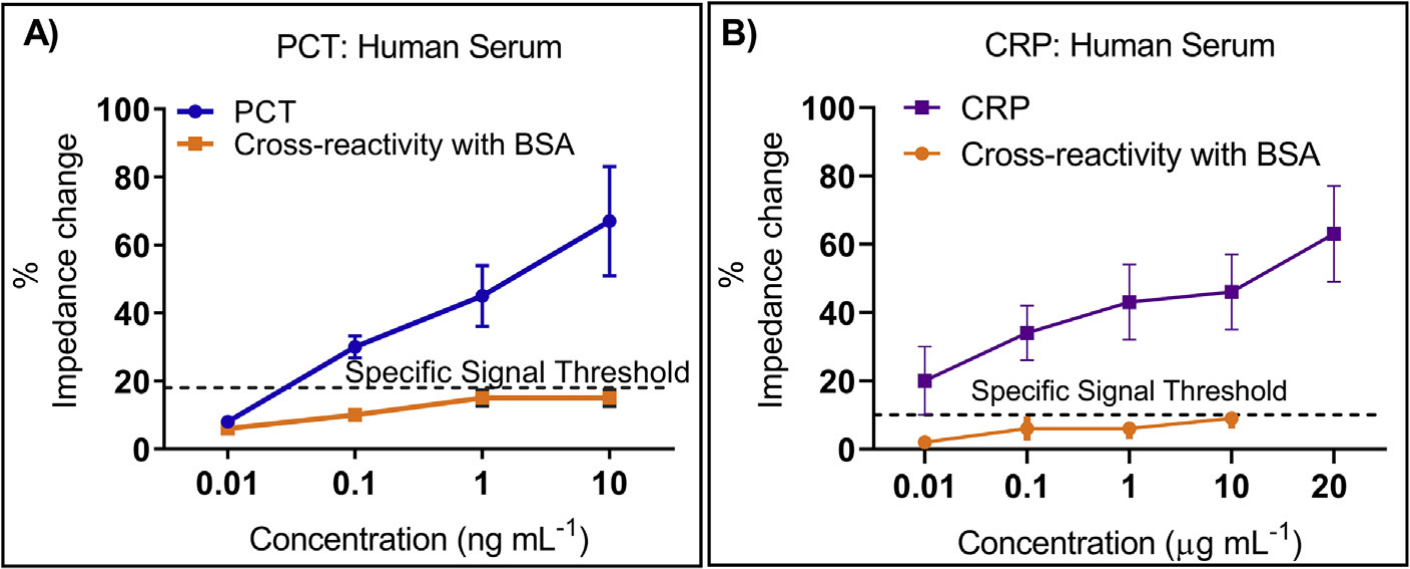
A) Selective response of PCT antibody immobilized surface evaluated with non-specific BSA in human serum represented as percentage change in impedance. (B) Selective response of CRP antibody immobilized surface evaluated with non-specific BSA in human serum.

The key feature of a reliable biosensor is the ability to specifically react only to the target analyte. This feature makes the sensor robust against false positive response, thereby, improving overall sensor performance. On successfully establishing a sensitive response from the calibration dose response, the sensor was challenged with a complex non-specific protein mixture of bovine serum albumin (BSA). The cross-reactivity of PCT specific capture antibody was tested with varying concentrations of BSA diluted in human serum as seen in Fig. 8A in the absence of target PCT protein. The signal in response to BSA showed a maximum of 15% change in impedance wherein the actual PCT response was approximately five times higher. Specific signal threshold (SST) was calculated to distinguish signal from noise by measuring three times standard deviation of blank dose and was plotted at 18%. As seen in Fig. 8A, PCT specific antibody displayed the sensor’s response to BSA which was below the SST, indicating specific response to target PCT biomolecule. Similarly, the specificity of CRP as the specific capture probe antibody was evaluated with BSA, as seen in Fig. 8B. The signal response obtained for BSA was a maximum of 10% change in impedance as compared to the specific response of CRP with a maximum change of 63%. Furthermore, non-specific response of BSA falls well within the SST established at 10%, confirming the specificity of the biosensor to target CRP. Table 1 compares the performance of the developed biosensor with similar reported biosensors for PCT and CRP detection in various buffers. It can be observed that the analytical performance such as limit of detection, response time and sample volume of the developed biosensor was better compared to similar electrochemical biosensors owing to the distinct semiconducting properties coupled with unique frequency signatures through EIS.

**Table 1:** Analytical performance comparison of various electrochemical biosensors.

Biomolecule	Buffer	LOD	Technique	Redox Label	Reference
PCT	Human serum	12.5 ng mL^−1^	EIS	Yes	[24]
	Human serum	0.10 ng mL^−1^	EIS	No	[26]
	Phosphate Buffer Saline	9.90 ng mL^−1^	SPR	–	[25]
	Human serum	0.10 ng mL^−1^	EIS	No	**This work**
	Whole blood	0.10 ng mL^−1^	EIS	No	**This work**
CRP	Human serum	2.00 μg mL^−1^	SPR	–	[27]
	Human serum	3.125 μg mL^−1^	EIS	Yes	[28]
	Phosphate Buffer Saline	0.01 μg mL^−1^	EIS	–	[29]
	Human serum	0.10 μg mL^−−1^	EIS	No	**This work**
	Whole blood	0.10 μg mL^−−1^	EIS	No	**This work**

## 4 Conclusion

In summary, we have devised a unique strategy using favorable intrinsic properties of ZnO electrode interface in conjunction with frequency optimization through EIS for the detection of PCT and CRP in human serum and whole blood for early detection of sepsis. The antibody-antigen binding interaction at the electrode-electrolyte interface was characterized by charge modulation within the electrical double layer for sensitive and selective detection of target analyte. Empirical frequency signatures were used to map the response of target specific biomarkers to develop the dual marker biosensor for PCT and CRP. The developed biosensor exhibited excellent sensitivity with a detection limit of 0.10 ng mL^−1^ for PCT and 0.10 μg mL^−1^ for CRP in human serum. The dual marker biosensor demonstrated highly specific response with minimal cross-reactivity to nonspecific protein molecules in human serum. The point-of- care biosensor also displayed feasibility of detection in complex body fluid such as whole blood with detection limit of 0.10 ng mL^−1^ for PCT and 0.10 μg mL^−1^ for CRP. The platform’s ability to detect both PCT, an early marker and CRP, a late onset biomarker of sepsis can help to determine the severity of infection and aid physicians in monitoring a patient’s response to treatment. In this work, we leverage the advantages of EIS in developing a robust sensor interface through frequency tuning to achieve specific response to differentiate biomarkers thus, enabling a dual marker biosensing system. The frequency signature pertaining to individual biomarkers is reflective of inherent charge distribution of the biomolecule wherein maximum signal response is achieved. We envision such a sensing system would allow medical practitioners to facilitate targeted interventions, thereby, offering immediate prognostic approach as the cornerstone to deliver successful treatment for sepsis and avert severe morbidity and mortality.

## Declaration of competing interest

The authors declare the following financial interests/personal relationships which may be considered as potential competing interests: Drs. Shalini Prasad and Sriram Muthukumar have a significant interest in Enlisense LLC, a company that may have a commercial interest in the results of this research and technology. The potential individual conflict of interest has been reviewed and managed by The University of Texas at Dallas, and played no role in the study design; in the collection, analysis, and interpretation of data; in the writing of the report, or in the decision to submit the report for publication.
