# Supplementary material for: Non-faradaic electrochemical impedimetric profiling of procalcitonin and C-reactive protein as a dual marker biosensor for early sepsis detection
Source: Anal Chim Acta X. 2019 Oct 3;3:100029. doi: 10.1016/j.acax.2019.100029 (PMC7587050; doi:10.1016/j.acax.2019.100029)
Supplement: Multimedia component 1 [file mmc1.docx]

**Non-faradaic electrochemical impedimetric profiling of Procalcitonin and C-reactive protein as a dual marker biosensor for early sepsis detection**

Ambalika Sanjeev Tanak^1^, Badrinath Jagannath^1^, Yashaswee Tamrakar^1^, Sriram Muthukumar^2^, Shalini Prasad^1^

1. Department of Bioengineering, The University of Texas at Dallas, Richardson, TX, USA 75080
2. EnLiSense LLC, 1813 Audubon Pondway, Allen, TX, USA 75013

Corresponding Author: Shalini Prasad

Address: 800 W. Campbell Rd. BSB 11,

Richardson, TX, USA 75080

Email: [shalini.prasad@utdallas.edu](mailto:shalini.prasad@utdallas.edu)

Phone: 972-883-4247

**Supplementary**

**
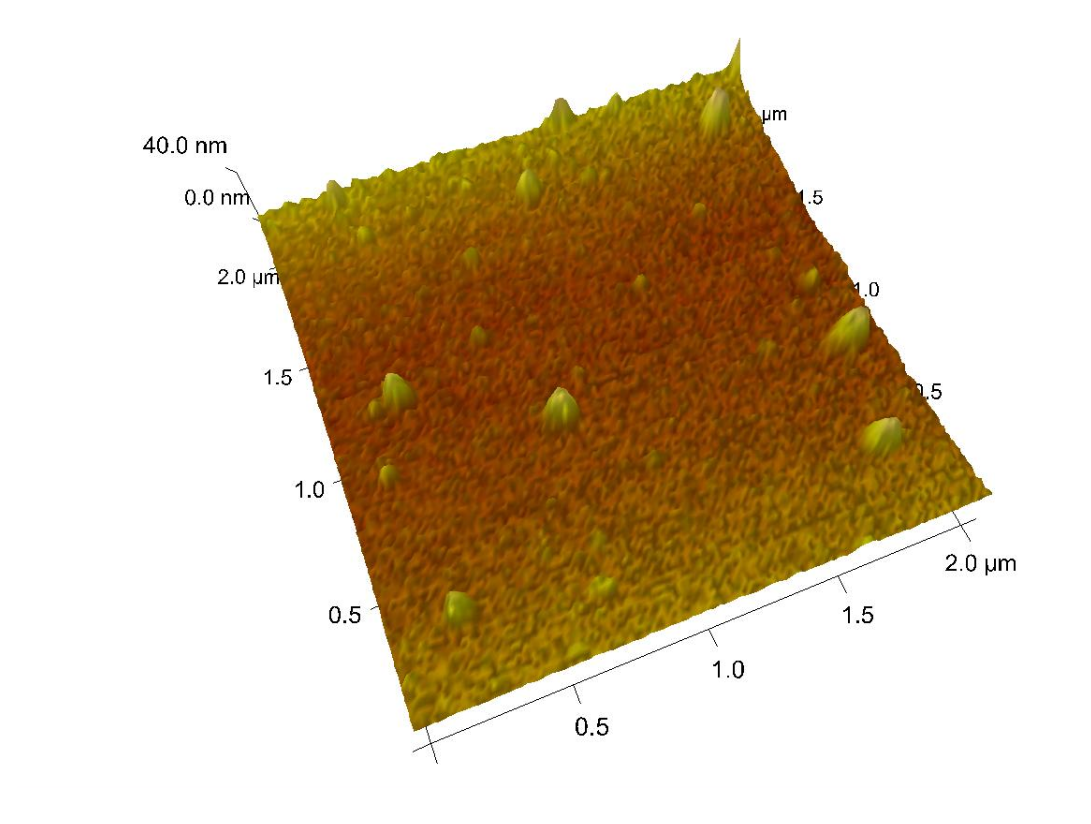
**

Figure S1: AFM image of the ZnO deposited thin film on sensor surface.


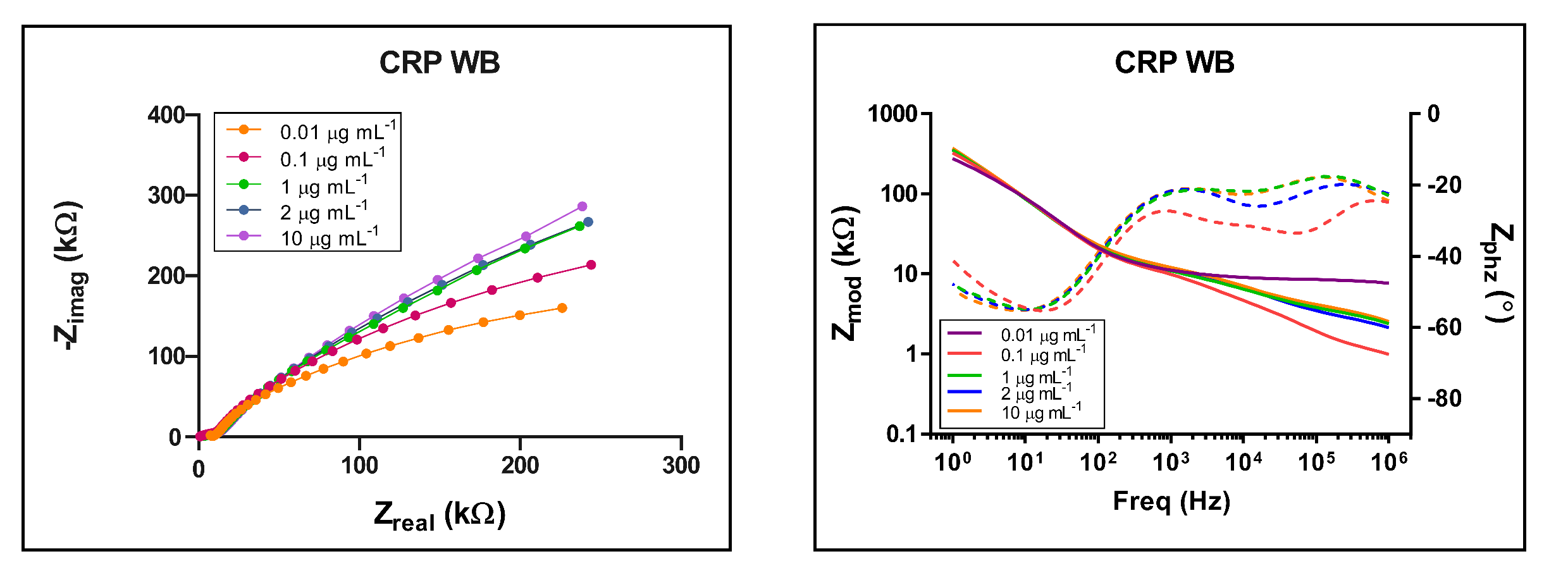


Figure S2: Nyquist plot of CRP spiked in whole blood


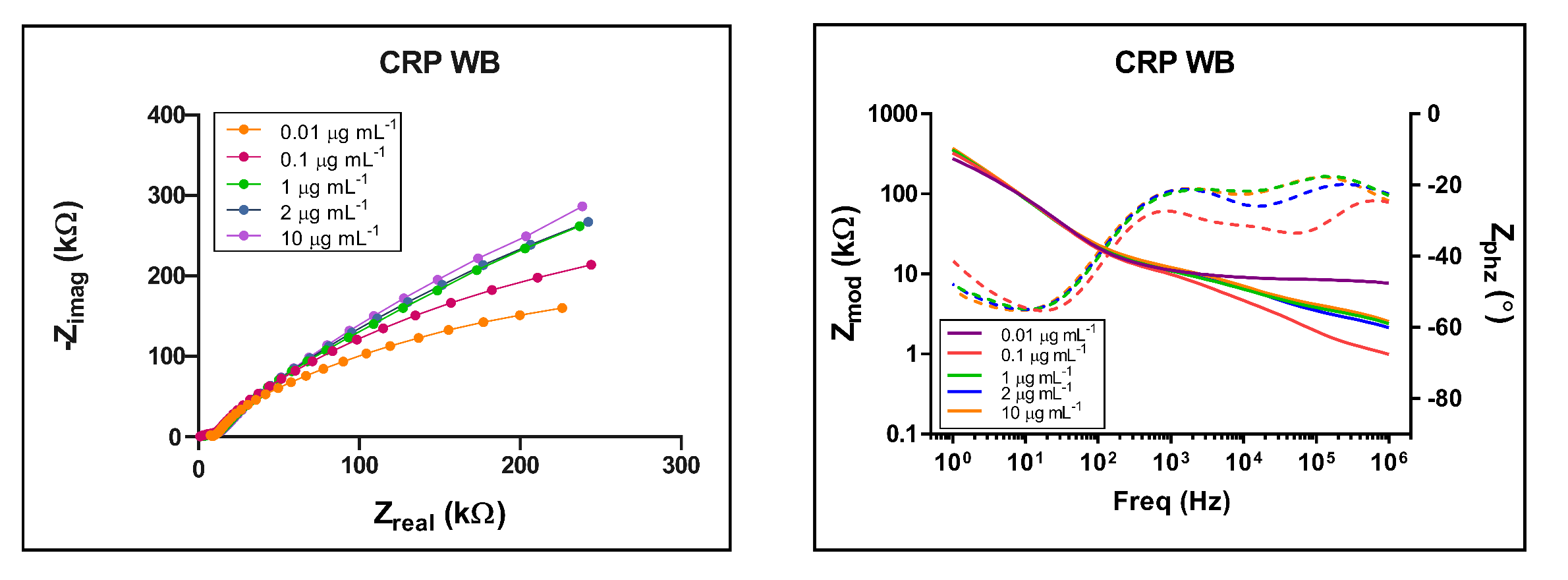


Figure S3: Bode phase and magnitude plot for CRP spiked in whole blood


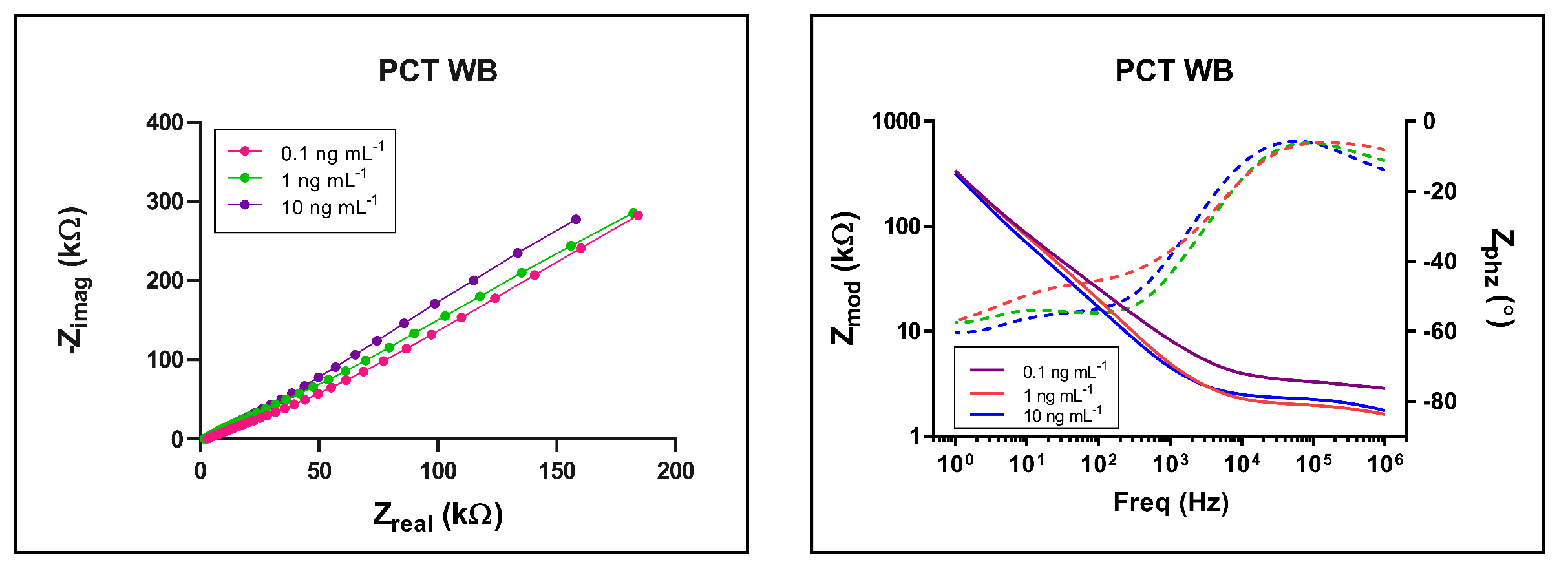


Figure S4: Nyquist plot for PCT spiked in whole blood


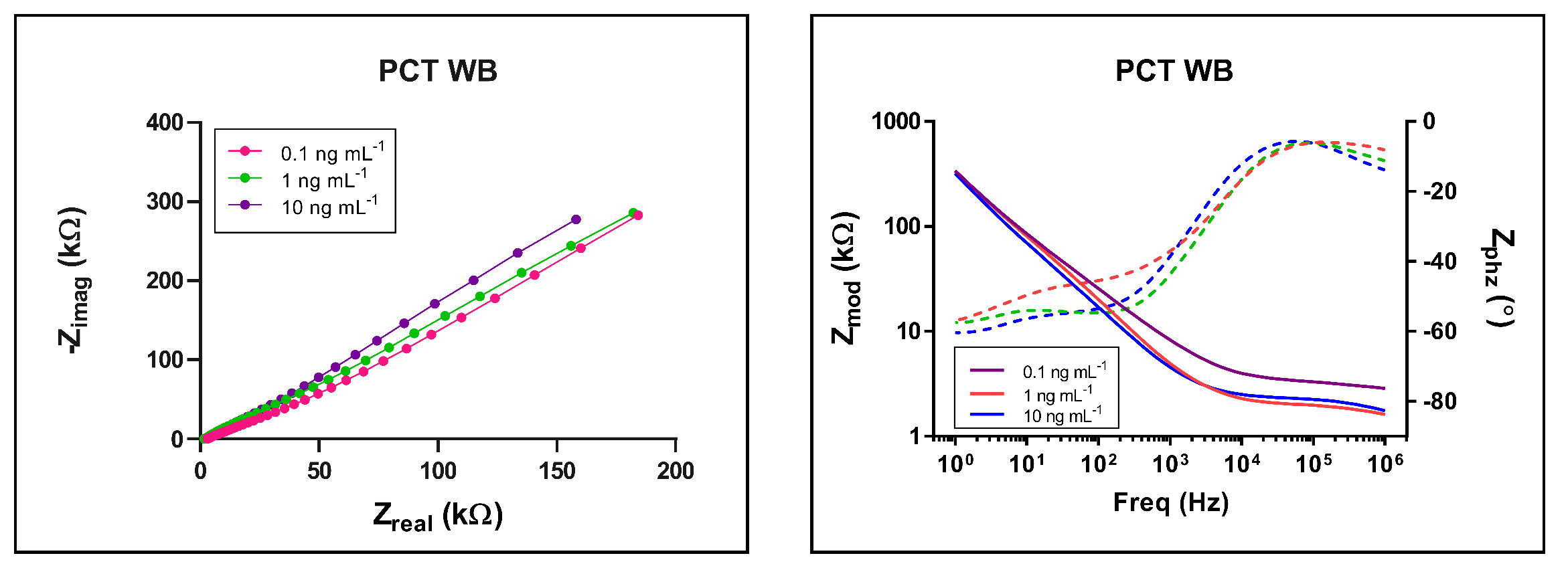


Figure S5: Bode phase and magnitude plot for CRP spiked in whole blood

| **Biofluid** | **Analytical Performance** | **PCT** | **CRP** |
| --- | --- | --- | --- |
| Human serum | Limit of Detection | 0.1 ngmL^-1^ | 0.1 µgmL^-1^ |
|  | Detection Range | 0.1-10 ngmL^-1^ | 0.1-20 µgmL^-1^ |
|  | Sensitivity | 8.33 Ω/ng mL^-1^ | 4.73 Ω/µgmL^-1^ |
| Whole Blood | Limit of Detection | 0.1 ng mL^-1^ | 0.1 µgmL^-1^ |
|  | Detection Range | 0.1-10 ng mL^-1^ | 0.1-10 µgmL^-1^ |
|  | Sensitivity | 0.024 Ω/ng mL^-1^ | 0.032 Ω/µg mL^-1^ |

Table S1. Analytical sensor performance metrics computed for the dual panel biosensing platform spiked in complex body fluids using EIS techniques
